# Emotional and Interactional Prosody across Animal Communication Systems: A Comparative Approach to the Emergence of Language

**DOI:** 10.3389/fpsyg.2016.01393

**Published:** 2016-09-28

**Authors:** Piera Filippi

**Affiliations:** Department of Artificial Intelligence, Vrije Universiteit BrusselBrussels, Belgium

**Keywords:** language evolution, musical protolanguage, prosody, interaction, turn-taking, arousal, infant-directed speech, entrainment

## Abstract

Across a wide range of animal taxa, prosodic modulation of the voice can express emotional information and is used to coordinate vocal interactions between multiple individuals. Within a comparative approach to animal communication systems, I hypothesize that the ability for emotional and interactional prosody (EIP) paved the way for the evolution of linguistic prosody – and perhaps also of music, continuing to play a vital role in the acquisition of language. In support of this hypothesis, I review three research fields: (i) empirical studies on the adaptive value of EIP in non-human primates, mammals, songbirds, anurans, and insects; (ii) the beneficial effects of EIP in scaffolding language learning and social development in human infants; (iii) the cognitive relationship between linguistic prosody and the ability for music, which has often been identified as the evolutionary precursor of language.

## Prosody in Human Communication

Whenever listeners comprehend spoken speech, they are processing sound patterns. Traditionally, studies on language processing assume a two-level hierarchy of sound patterns, a property called “duality of pattern” or “double articulation” (Hockett, 1960; Martinet, 1980). The first dimension is the concatenation of meaningless phonemes into larger discrete units, namely morphemes, in accordance to the phonological rules of the given language. At the next level, these phonological structures are formed into words and morphemes with semantic content and arranged within hierarchical structures (Hauser et al., 2002), according to morpho-syntactical rules. Surprisingly, this line of research has often overlooked prosody, the “musical” aspect of the speech signal, i.e., the so-called “suprasegmental” dimension of the speech stream, which includes timing, frequency spectrum, and amplitude (Lehiste, 1970). Taken together, these values outline the overall prosodic contour of words and/or sentences. According to the source–filter theory of voice production (Fant, 1960; Titze, 1994), vocalizations in humans -and in mammals more generally- are generated by airflow interruption through vibration of the vocal folds in the larynx (‘source’). The signal produced at the source is subsequently filtered in the vocal tract (‘filter’). The source determines the fundamental frequency of the call (*F0*), and the filter shapes the source signal, producing a concentration of acoustic energy around particular frequencies in the speech wave, i.e., the formants. Thus, it is important to highlight that in producing vocal utterances, speakers across cultures and languages modulate *both* segmental, and prosodic information in the signal. In humans, prosodic modulation of the voice affects language processing at multiple levels: linguistic (lexical and morpho-syntactic), emotional, and interactional.

### Linguistic Prosody

Prosody has a key role in word recognition, syntactic structure processing, and discourse structure comprehension (Cutler et al., 1997; Endress and Hauser, 2010; Wagner and Watson, 2010; Shukla et al., 2011). Prosodic cues such as lexical stress patterns specific to each natural language are exploited to segment words within speech streams (Mehler et al., 1988; Cutler, 1994; Jusczyk and Aslin, 1995; Jusczyk, 1999; Johnson and Jusczyk, 2001; Curtin et al., 2005). For instance, many studies of English have indicated that segmental duration tends to be longest in word-initial position and shorter in word-final position (Oller, 1973). Newborns use stress patterns to classify utterances into broad language classes defined according to global rhythmic properties (Nazzi et al., 1998). The effect of prosody in word processing is distinctive in tonal languages, where F0 variations on the same segment results in totally different meanings (Cutler and Chen, 1997; Lee, 2000). For instance, the Cantonese consonant-vowel sequence [si] can mean “poem,” “history,” or “time,” based on the specific tone in which it is uttered.

Prosodic variations such as phrase-initial strengthening through pitch rise, phrase-final lengthening, or pitch discontinuity at the boundaries between different phrases mark morpho-syntactic connections within sentences (Soderstrom et al., 2003; Johnson, 2008; Männel et al., 2013). These prosodic variations mark phrases within sentences, favoring syntax acquisition in infants (Steedman, 1996; Christophe et al., 2008) and guiding hierarchical or embedded structure comprehension in continuous speech in adults (Müller et al., 2010; Langus et al., 2012; Ravignani et al., 2014b; Honbolygo et al., 2016). Moreover, these prosodic cues enable the resolution of global ambiguity in sentences like “flying airplanes can be dangerous” – which can mean that *the act* of flying airplanes can be dangerous or that the objects *flying airplanes* can be dangerous – or “I read about the repayment with interest,” where “with interest” can be directly referred to the act of reading or to the repayment. Furthermore, sentences might be characterized by local ambiguity, i.e., ambiguity of specific words, which can be resolved by semantic integration with the following information within the same sentence, as in “John believes Mary implicitly” or “John believes Mary to be a professor.” Here, the relationship between “believes” and “Mary” depends on what follows. In the case of both global and local ambiguity, prosodic cues to the syntactical structure of the sentence aid the understanding of the utterance meaning as intended by the speaker (Cutler et al., 1997; Snedeker and Trueswell, 2003; Nakamura et al., 2012).

Prosodic features of the signal are used to mark questions (Hedberg and Sosa, 2002; Kitagawa, 2005; Rialland, 2007), and in some languages, prosody serves as a marker of salient (Bolinger, 1972) or new (Fisher and Tokura, 1995) information. Consider for instance, “MARY gave the book to John” vs. “Mary gave the book to JOHN,” in which the accented word is the one the speaker wants to drive the listener’s attention to in the conversational context.

### Emotional Prosody In Humans

The prosodic modulation of the utterance can signal the emotional state of the speaker, independently from her/his intention to express an emotion. Research suggests that specific patterns of voice modulation can be considered a “biological code” for both linguistic and paralinguistic communication (Gussenhoven, 2002). Indeed, physiological changes might cause tension and action of muscles used for phonation, respiration, and speech articulation (Lieberman, 1967; Scherer, 2003). For instance, physiological variations in an emotionally aroused speaker might cause an increase of the subglottal pressure (i.e., the pressure generated by the lungs beneath the larynx), which might affect voice amplitude and frequency, thus expressing his/her emotional state. Crucially, in cases of emotional communication, prosody can prime or guide the perception of the semantic meaning (Ishii et al., 2003; Schirmer and Kotz, 2003; Pell, 2005; Pell et al., 2011; Newen et al., 2015; Filippi et al., 2016). Moreover, the expression of emotions through prosodic modulation of the voice, in combination with other communication channels, is crucial for affective and attentional regulation in social interactions both in adults (Sander et al., 2005; Schore and Schore, 2008) and infants (see section “EIP in Language Acquisition” below).

### Prosody for Interactional Coordination In Humans

A crucial aspect of spoken language is its interactional nature. In conversations, speakers typically use prosodic cues for interactional coordination, i.e., implicit turn-taking rules that aid the perception of who is to speak next and when, predicting the content and timing of the incoming turn (Roberts et al., 2015). The typical use of a turn-taking system might explain why language is organized into short phrases with an overall prosodic envelope (Levinson, 2016). Within spoken interactions, prosodic features such as low pitch or final word lengthening are used for turn-taking coordination, determining the rhythm of the conversations among speakers (Ward and Tsukahara, 2000; Ten Bosch et al., 2005; Levinson, 2016). These prosodic features in the signal are used to recognize opportunities for turn transition and appropriate timing to avoid gaps and overlaps between speakers (Sacks et al., 1974; Stephens and Beattie, 1986). Wilson and Wilson (2005) suggested that both the listener and the speaker engage in an oscillator-based cycle of readiness to initiate a syllable, which is at a minimum in the middle of syllable production, at the point of greatest syllable sonority, and at a maximum when the prosodic values of the syllable lessen, typically in the final part of the syllable. The listener is entrained by the speaker’s rate of syllable production, but her/his cycle is counterphased to the speaker’s cycle. Therefore, the listener will be able to take turn in speaking if s/he detects that the speaker is not initiating a new cycle of syllable production. In accordance to this model, Stivers et al. (2009) provided evidence for biologically rooted timings in replying to speakers on the base of prosodic features in the signal, a finding that is indicative of a strong universal basis for turn-taking behavior. Specifically, this study provides evidence for a similar distribution of response offsets (unimodal peak of response within 200 ms of the end of the utterance) across conversations in ten languages drawn from traditional indigenous communities to major world languages. The authors observed a general avoidance of overlapping talk and minimal silence between conversational turns across all tested languages.

### A Comparative Approach to Emotional and Interactional Prosody

**FIGURE 1:**
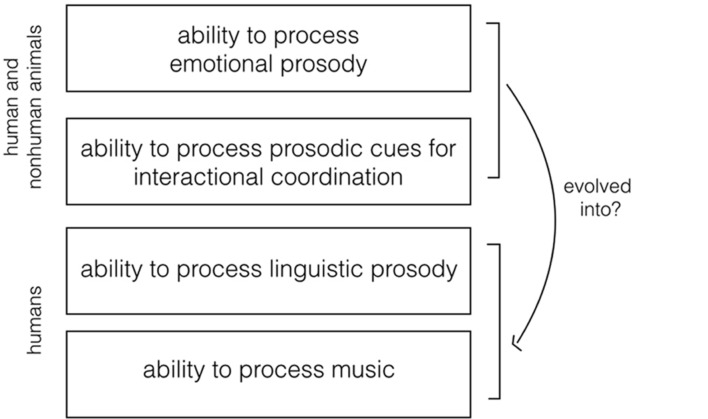
**Visual representation of the research hypothesis**. The ability to process acoustic prosody in emotional communication and in interactional coordination is widespread across animal taxa. Here, I hypothesize that this ability evolved into the ability to process linguistic prosody and music in humans.

Given the centrality of prosody in spoken communication, it is worth addressing the adaptive role of prosody on both an evolutionary and a developmental level. Here, I hypothesize that prosodic modulation of the voice marking emotional communication and interactional coordination (hereafter EIP, emotional and interactional prosody), as we observe it nowadays across multiple animal taxa, evolved into the ability to modulate prosody for language processing – and might have played an important role in the emergence of music (**Figure 1**) (Phillips-Silver et al., 2010; Bryant, 2013; Zimmermann et al., 2013). In support of this hypothesis, within a comparative approach, I will review studies on the adaptive use of prosodic modulation of the voice for emotional communication and interactional coordination in animals.

Importantly, following Morton (1977) and Owren and Rendall (1997), I aim to address the behavioral and functional effects of emotional vocalizations in animals, as conveyed by their prosodic characteristics and by the interactional dynamics of communication act. Therefore, I will adopt the very basic, but fundamental assumption that the prosodic structure of calls (which reflects the physiological/emotional state of the signaler) and call-answer dynamics induce nervous-system and physiological responses in the receiver. For instance, a call might induce an increased level of emotional arousal or of attention. These physiological responses might trigger specific types of behaviors in the listeners, for instance escape or physical approaching (Nesse, 1990; Frijda, 2016). Ultimately, these behaviors are the immediate functional effect of the communication act (Owren and Rendall, 2001; Rendall et al., 2009).

A crucial dimension, constitutive of a multiple communicative behaviors across animal species, is interactional coordination. Examples of interactional coordination are widespread across animal classes, including unrelated taxa. This suggests that this ability has evolved independently in a number of species under similar selective pressures (Ravignani, 2014). There are three main types of interactional coordination in animal acoustic communication: choruses, antiphonal calling, and duets (Yoshida and Okanoya, 2005). In choruses, males simultaneously emit a signal for sexual advertisement or as an anti-predator defensive behavior. Antiphonal calling occurs when more than two members of a group exchange calls within an interactive context. Duets occur when members of a pair (e.g., sexual mates, caregiver-juvenile) exchange calls within a precise time window. Importantly, the modulation of the prosodic features of the vocal signals is key to coordinating these communicative behaviors.

Based on Tinbergen (1963), in order to grasp an integrative understanding of animal vocal communication, I will go through four levels of description: mechanisms, functional effects (**Table 1**), phylogenetic history, and ontogenetic development. Two strands of analysis are relevant in the context of comparative investigation on the adaptive advantages of prosody in relation to the origins of language: (a) research on the evolutionary ‘homologies,’ which provides information on the phylogenetic traits that humans and other primates share with their common ancestor; (b) investigations on “analogous” traits, aimed at finding the evolutionary pressures that guided the emergence of the same biological traits that evolved independently in phylogenetically distant species (Gould and Eldredge, 1977; Hauser et al., 2002). As to the ontogenetic level of explanation, I will review empirical data on the beneficial effects of EIP for the development of social and vocal learning skills in multiple animal species.

**Table 1:** Overview of the functional effects of emotional and interactional prosody across diverse animal taxa.

		Insects	Anurans	Birds	Non-human mammals	Non-human primates	Humans
Emotional prosody		Expression of the signaler’s physiological state					Expression of the signaler’s physiological state, affective regulation of interpersonal interactions

	Chorus	Sexual advertisement, anti-predator behavior		Social bonding, synchronization of activities and group or territory defense	[Not reported]	[Not reported]	Social entrainment, group cohesion, cooperation
	
Prosody for interactional coordination in auditory communication	Antiphonal calling	[Not reported]		Aggressive/submissive signaling in territorial contests	Spatial location, social bonding, identity signaling	Group cohesion	[Not reported]
	
	Duet	Sexual advertisement	Sexual advertisement, male–male competition	Adults: pair bonding, spacing of males, reunification of separated mates Tutor-juvenile: song learning	Sexual advertisement [reported only in Cape-mole rats]	Adults: pair bonding, territory and resource defense Caregiver-juvenile: interpersonal bonding, social development, vocal development	Adults: inter-individual affective regulation Caregiver-Infant: socio-cognitive development, sense of agency, language development

Within this line of research, it is important to highlight that extensive research has identified the evolutionary precursor of language in a general ability to produce music (Brown, 2001; Mithen, 2005; Patel, 2006; Fitch, 2010, 2012). There are at least two orders of argumentation supporting the hypothesis that aspects of musical processing were involved in human language evolution: (a) research on the cognitive link between music and verbal language processing; (b) comparative data on animal communication systems, suggesting that this ability, already in place in different primate as well as in many non-primate species, might have evolved into an adaptive ability in the first hominins. Based on the reviews of (a) and (b), I propose to identify the emotional and interactional functions of prosody as dimensions that are sufficient to an account for the “musical” origins of language. This conceptual operation will provide a parsimonious account for the investigation of the origins of language as well as of language acquisition at a developmental level, keeping this research close to both ethological and cognitive principles of explanation.

## Musical Origins of Language: Revisiting Darwin’S Hypothesis

A close look to the empirical studies on animal communication reveals how EIP is widespread across a broad range of animal taxa. A comparative investigation will provide us with relevant information on the adaptive valence, and therefore on the evolutionary role, of such crucial dimensions in the domain of animal communication. Darwin provides an important insight on this topic:

Primeval man, or rather some early progenitor of man, probably first used his voice in *producing true musical cadences, that is in singing*, as do some of the gibbon-apes at the present day; and we may conclude, from a wide-spread analogy, that this power would have been especially exerted during the courtship between sexes, – would have expressed various emotions, such as love, jealousy, triumph, – and would have served as a challenge to rivals.

(Darwin, 1871, pp. 56–57; my emphasis).

Darwin’s hypothesis that early humans were *singing*, as gibbons do today, has called for a comparative investigation into the ability to make “music” as a precursor of language (Rohrmeier et al., 2015). In order to gain a clearer understanding of the adaptive value of *musical* vocalizations in animals, and of its adaptive role for the emergence of human language, we need to examine: (i) to what extent it is correct to attribute musical abilities to non-human animals, and (ii) whether the ability to process EIP, rather then a general ability for music in non-human animals, can be considered an adaptive prerequisite necessary for the emergence of human language. I believe that making the distinction between a general aptitude for music and the use of EIP, might improve the investigation of the origins of language. This line of investigation will shed light on the adaptive role of EIP for the emergence of language, and perhaps of the ability for music itself in both human and non-human animals.

The question, then, is: Are gibbons, and non-human animals in general, able to make music in a way that is comparable to humans? Recent research has shown that birds, monkeys, and humans share the predisposition to distinguish consonant vs. dissonant music (Hulse et al., 1995; Izumi, 2000; Sugimoto et al., 2009). Moreover, studies suggest that rhesus macaques, *Macaca mulatta* (Wright et al., 2000), rats, *Rattus norvegicus* (Blackwell and Schlosberg, 1943), and dolphins, *Tursiops truncatus* (Ralston and Herman, 1995) are able to recognize two melodies as the “same” melody even when transposed one octave up or down. Songbirds, which in contrast miss this ability, have been shown to rely on absolute frequency over relative pitch within a scale (Cynx, 1995; Hoeschele et al., 2013). Furthermore, as Patel (2010) suggests, birdsong has a rhythm that, despite violating human metric conventions, is nonetheless stable and internally consistent. Recent research has also established that some parrot species (*Cacatua galerita and Melopsittacus undulatus*) and a California sea lion (*Zalophus californianus*) are able to extract the pulse from musical rhythm, moving along with it (Fitch, 2013 for a review). Hence, we can accept that a biological inclination toward the ability for music is also present, to a certain extent, in non-human animals (Doolittle and Gingras, 2015; Fitch, 2015; Hoeschele et al., 2015).

However, non-human animals’ ability to modulate sounds in courtship or rivalry contexts, which Darwin identified as a precursor of language, might be described, more parsimoniously, as an instance of EIP. Here, I suggest that the ability to modulate prosody in emotional communication and within turn-taking contexts (rather than the ability for music), as enough to describe the emergence of vocal utterances in the early *Homo*. Darwin’s hypothesis may thus be updated in light of contemporary research and read in the following terms: the first hominins communicated exploiting prosody for emotional expression and communicative coordination. As I will clarify in the following sections, extensive research indicates that in different animal species the ability to vary prosodic features in the voice, in conjunction with the ability to coordinate sound production with others – expressing emotions, and possibly triggering emotional reactions – has an adaptive value. This use of prosody has positive effects in relation to sexual partner attraction, territory defense, group cohesion, parental care (Searcy and Andersson, 1986). Thus, the investigation of prosodic modulation of the voice provides an excellent, and surprisingly overlooked paradigm for a comparative approach addressing the adaptive features grounding the emergence of language. In the next sections, I will review studies reporting on EIP in non-human primates, non-primate mammals, birds, insects, and anurans.

### EIP in Non-human Primates

The ability to modulate the prosodic features of a signal can be considered a homologous trait, i.e., a trait that humans and other primates share with their common ancestor. Experiments conducted both in the field and in captivity suggest that several species of prosimians and anthropoids are able to modulate spectro-temporal features of a call (frequency, tempo, and amplitude) as noise-induced vocal modifications (Hotchkin and Parks, 2013 for an extensive review). Research on chimpanzees’ (*Pan troglodytes*) panthoots, a type of long-distance calls emitted while traveling or in the presence of abundant food sources, reveals individual and contextual modulation of the prosodic structure of this call (Notman and Rendall, 2005). De la Torre and Snowdon (2002) found that also pygmy marmosets, *Cebuella pygmaea*, adjust the frequency and temporal structure of their contact calls in a way appropriate to the frequency distortion effects of the habitats where they are located in order to maintain the acoustic structure of the long distance vocalization.

Studies provide evidence on arousal-related modulation of the call structure in non-human primates (Morton, 1977; Briefer, 2012). Specifically, it has been shown that high call rate (tempo), number of calls, and elevated fundamental frequency range correlate positively with high levels of arousal in chimpanzees, *Pan troglodytes* (Riede et al., 2007), squirrel monkeys, *Saimiri sciureus* (Fichtel et al., 2001), bonnet macaques, *Macaca radiata* (Coss et al., 2007), vervet monkeys, *Macaca mulatta* (Seyfarth et al., 1980), rhesus monkeys, *Chlorocebus pygerythrus* (Hauser and Marler, 1993; Jovanovic and Gouzoules, 2001; Hall, 2009), baboons, *Papio papio* (Rendall et al., 1999; Seyfarth and Cheney, 2003), mouse lemurs, *Microcebus* spp. (Zimmermann, 2010), tree shrews, *Tupaia belangeri* (Schehka et al., 2007). It is important to stress that the modulation of these acoustic features of the signal derives from arousal-based physiological changes, thus these modulations are not under the voluntary control of the signaler. For instance, emotionally induced changes in muscular tone and coordination can affect the tension in the vocal folds, and consequently the fundamental frequency range of the vocalization and the voice quality of the caller (Rendall, 2003). Crucially, although the transmission of the emotional content of the signal is not intentional, the receivers are nonetheless sensitive to it, and are able to perceive, for instance, the level of urgency of the situation in which the call is produced, behaving in the most adaptive way (Zuberbühler et al., 1999; Seyfarth and Cheney, 2003). Further research is required to investigate whether different levels of arousal are encoded in (or decoded from) the structure of the interactive calls between conspecifics (Filippi et al., submitted), and whether the dynamics of alternate calling affects the emotional or attentive state of the signalers themselves.

Evidence suggests that non-human primates can coordinate the production of a signal with the vocal behavior of a mate or of other individuals of a group, modulating the acoustic features of vocalizations for communicative purposes. For instance, the ability for antiphonal calling, i.e., to flexibly respond to conspecifics in order to maintain contact between group members, has been reported in recent work conducted across prosimians, monkeys, and lesser apes: chimpanzees, *Pan troglodytes* (Fedurek et al., 2013), barbary macaques, *Macaca sylvanus* (Hammerschmidt et al., 1994), Campbell’s monkeys, *Cercopithecus campbelli* (Lemasson et al., 2010), Diana monkeys, *Cercopithecus diana* (Candiotti et al., 2012), pygmy marmosets, *Cebuella pygmaea* (Snowdon and Cleveland, 1984), common marmosets, *Callithrix jacchus* (Miller et al., 2009), cotton-top tamarins, *Saguinus oedipus* (Ghazanfar et al., 2002), squirrel monkeys, *Saimiri sciureus* (Masataka and Biben, 1987), vervet monkeys, *Macaca mulatta* and *Chlorocebus pygerythrus* (Hauser, 1992), geladas, *Theropithecus gelada* (Richman, 2000) and Japanese macaques, *Macaca fuscata* (Sugiura, 1993; Lemasson et al., 2013). These so-called antiphonal vocalizations are guided by a sort of “turn taking” conversational rule system employed within an interactive and reciprocal dynamic between the calling individuals. Versace et al. (2008) found that cotton top tamarins, *Saguinus oedipus* can detect and wait for silent windows to vocalize. Call alternation in monkeys promotes social bonding and keeps the members of a group in vocal contact when visual access is precluded.

Turn-taking duet-like activities have been reported in caregiver-juvenile pairs in gibbons (Koda et al., 2013) and marmosets (Chow et al., 2015). In both species, caregivers interact with their juveniles, engaging in time-coordinated vocal feedback. This behavior scaffolds the development of turn-taking and social competences in the juvenile marmosets, *Callithrix jacchus* (Chow et al., 2015), and seems to enhance vocal development in juvenile gibbons, *Hylobates agilis agilis* (Koda et al., 2013). Vocal duets in male-female pairs have been reported in: gibbons, *Hylobates* spp (Geissmann, 2000a), lemurs, *Lepilemur edwardsi*; (Méndez-Cárdenas and Zimmermann, 2009), common marmosets, *Callithrix jacchus* (Takahashi et al., 2013), the coppery titi, *Callicebus cupreus* (Müller and Anzenberger, 2002), squirrel monkeys, *Saimiri* spp. (Symmes and Biben, 1988), Campbell’s monkeys, *Cercopithecus campbelli* (Lemasson et al., 2011), siamangs *Hylobates syndactylus* (Haimoff, 1981; Geissmann and Orgeldinger, 2000). Duets constitute a remarkable instance of interactional prosody, where members of a pair coordinate their sex-specific calls, effectively *composing* a single ‘song’ with two voices. Duets are interactive processes that involve time- and pattern-specific coordination among vocalizations flexibly exchanged between two individuals. Such a level of vocal coordination requires extensive practice over a long period of time. It seems that this investment strengthens the bond between the partners, since the quantity of duets performed is positively correlated with the pair bonding quality (measured by with grooming practice and physical proximity). In turn, the strength of the pair bonding also has positive adaptive effects on the management of parental care, territory defense, or foraging activities (Geissmann, 2000b; Geissmann and Orgeldinger, 2000; Müller and Anzenberger, 2002; Méndez-Cárdenas and Zimmermann, 2009).

From this set of studies we can infer that non-human primates possess the ability to process EIP, which is linked to group cohesion, territory defense, pair bonding, parental care, and social development. In conclusion, comparative review of studies on EIP in primates supports the hypothesis that these abilities have a functional role, and can thus be considered adaptive “homologous” traits in non-human primates.

### EIP in Non-primate Mammals

Comparative research on non-primate mammals addressed the ability to modulate prosodic features of the voice, which express different levels of emotional arousal, and are used in interactive communications. These studies, focused on traits that are analogous in humans and non-primate mammals, are crucial within a comparative frame of research, as they may shed light on the selective pressures favoring the emergence of the human ability to process prosody as cue to language comprehension and maybe also of the human inclination for music.

Evidence has been reported on the ability to modulate the prosodic features of the vocal signals in several non-primate mammals: bottlenose dolphins, *Tursiops truncatus* (Buckstaff, 2004), humpback whales, *Megaptera novaeangliae* (Doyle et al., 2008), killer whales, *Orcinus orca* (Holt et al., 2009, 2011), right whales, *Eubalaena glacialis* (Parks et al., 2007, 2011), free-tailed bat, *Tadarida brasiliensis* (Tressler and Smotherman, 2009), mouse-tailed bat, *Rhinopoma microphillum* (Schmidt and Joermann, 1986), Californian ground squirrel, *Spermophilus beecheyi* (Rabin et al., 2003), and domestic cats, *Felis catus* (Nonaka et al., 1997). Only little attention has been devoted to the emotional content of calls in the species mentioned above. However, recent research conducted on giant pandas, *Ailuropoda melanoleuca* (Stoeger et al., 2012) and on African elephants, *Loxodonta africana* (Soltis et al., 2005b; Stoeger et al., 2011) provides evidence that in mammals high levels of arousal can be expressed through specific acoustic features in the signal, namely: noisy and aperiodic segments, increased call duration and elevated fundamental frequency. The effective expression and perception of emotional arousal may allow individuals to respond appropriately, based on the degree of urgency or distress encoded in the call. Thus, the ability to process these calls correctly may be crucial for survival under natural conditions.

In addition, studies indicate that, in the case of conflicts or separation from the group and when visual cues are not available, the following species of mammals produce antiphonal calls to signal their identity or spatial location: African elephants, *Loxodonta africana* (Soltis et al., 2005a), Atlantic spotted dolphins, *Stenella frontalis* (Dudzinski, 1998), bottlenose dolphins, *Tursiops truncatus* (Janik and Slater, 1997; Kremers et al., 2014), white-winged vampire bats, *Diaemus youngi* (Carter et al., 2008, 2009; Vernes, 2016), horseshoe bats, *Rhinolophus ferrumequinum nippon* (Matsumura, 1981), killer whales, *Orcinus orca* (Miller et al., 2004), sperm whales, *Physeter macrocephalus* (Schulz et al., 2008), and naked mole-rats, *Heterocephalus glaber* (Yosida et al., 2007). Individuals in all these species alternate calls, following specific patterns of response timing to maintain group cohesion and bonding relationships. Furthermore, vocal duets have been reported in Cape-mole rats, *Georychus capensis* (Narins et al., 1992). Members of this species alternate seismic signals (generated by drumming their hind legs on the burrow floor) to attract sexual mates.

In sum, the studies reviewed in this section indicate that the ability to process EIP is present also in non-primate mammals, where it might have evolved as adaptive “analogous traits,” i.e., under the same selective pressures (group cohesion, territory defense, pair bonding, parental care) that triggered its emergence in primates.

### EIP in Birds

The study of mechanisms and processes underlying EIP in birds has revealed multiple analogous traits, i.e., strong evolutionary convergences, with vocal communication in humans. By shedding light on the selective pressures grounding the emergence of EIP in species that are phylogenetically distant, as it is the case for humans and birds, this line of research may enhance our understanding of the evolutionary path of the ability to process linguistic prosody (and perhaps also music) in humans.

Differently to mammalians, in birds, sounds are produced by airflow interruption through vibration of the labia in the syrinx (Gaunt and Nowicki, 1998). Modulation in bird vocalization is thought to originate predominantly from the sound source (Greenewalt, 1968), while the resonance filter shapes the complex time-frequency patterns of the source (Nowicki, 1987; Hoese et al., 2000; Beckers et al., 2003). For instance, songbirds are able to change the shape of their vocal tract, tuning it to the fundamental frequency of their song (Riede et al., 2006; Amador et al., 2008).

Importantly, variations in the prosodic features of the calls may be indicative of the emotional state of the signaler. The expression of arousal and/or emotional information through the modulation of prosody in birds has been shown in chickens, *Gallus gallus* (Marler and Evans, 1996), ring doves, *Streptopelia risoria* (Cheng and Durand, 2004), Northern Bald Ibis, *Geronticus eremita* (Szipl et al., 2014), black-capped chickadees, *Poecile atricapillus* (Templeton et al., 2005; Avey et al., 2011). The ability to process different levels of emotional arousal in bird vocalizations serve numerous functions including signaling type and degree of potential threats, dominance in agonistic contexts, or the presence of high quality food (Ficken and Witkin, 1977; Evans et al., 1993; Griffin, 2004; Templeton et al., 2005).

As to the interactional dimension of prosody, evidence for choruses has been reported in: Common mynas, *Acridotheres tristis* (Counsilman, 1974), Australian magpies, *Gymnorhina tibicen* (Brown and Farabaugh, 1991), and in black-capped chickadees, *Poecile atricapillus* (Foote et al., 2008). This activity has been shown to favor social bonding, synchronization of activities, and group or territory defense.

Research has described the capacity to modulate and coordinate vocal productions in antiphonal calling between individuals of different groups in European starlings, *Sturnus vulgaris* (Hausberger et al., 2008) and in nightingales, *Luscinia megarhynchos* (Naguib and Mennill, 2010). Crucially, Henry et al. (2015) found that prosodic features of vocal interactions in starlings are influenced by the immediate social context, the individual history, and the emotional state of the signaler. Camacho-Schlenker et al. (2011) suggest that in winter wrens, *Troglodytes troglodytes*, call exchanges among neighbors might have different aggressive/submissive values. Thus, these antiphonal calls can escalate in territorial contests, influencing females’ mate choice.

Multiple studies report duets in songbirds. Indeed, duets among sexual partners, which coordinated their phrases by alternation or overlap, are widespread among songbirds. As in non-human primates, they help to maintain pair bonds and are used to defend territories or resources. Duets have been reported in: fred-backed fairy-wrens, *Malurus melanocephalus* (Baldassarre et al., 2016; reviews: Langmore, 1998; Hall, 2009; Dahlin and Benedict, 2014). Notably, the capacity to coordinate the production of sounds with the vocalizations of a partner requires control over the modulation of phonation in frequency, tempo, and amplitude. Dilger (1953) suggests that in crimson-breasted barbets, *Psilopogon haemacephalus*, the coordination of two sexual mates in duetting could affect the production of reproductive hormones, thereby ensuring synchrony in the reproductive status of the breeding partners. Thus, the ability to coordinate or synchronize vocal sounds has an adaptive value that may have guided the evolution of song complexity and plasticity in songbirds (Kroodsma and Byers, 1991). Indeed, the ability to produce complex sequences of sounds is indicative of an individual’s capacity to memorize complex sequences and how fine a caller’s motor and neural control is over the sounds of the song (Searcy and Andersson, 1986; Langmore, 1998). This strong index of mental and physical skills is shown to be important in a mate choice context in zebra finches, *Taeniopygia guttata* (Neubauer, 1999) and Bengalese finches, *Lonchura striata* (Okanoya, 2004). Similarly, recent research conducted on humans suggest that women have sexual preferences during peak conception times for men that are able to create more complex sequences of sounds (Charlton et al., 2012; Charlton, 2014).

Importantly, both in humans and songbirds vocal learning has an interactive dimension. Interestingly, in both groups, the ability to alternate and coordinate vocalizations with conspecifics is acquired by interactive tutoring with adult conspecifics (Poirier et al., 2004; Feher et al., 2009; see section “EIP in Language Acquisition” below). Goldstein et al. (2003) argue that such convergence reveals that the social dimension is an important adaptive pressure that favored the acquisition of complex vocalizations in humans and songbirds (Syal and Finlay, 2011).

Taken together, studies reporting on EIP in songbirds support the hypothesis that the ability to modulate prosodic features of the calls, marking emotional expression and interactional coordination, can be identified as an analogous and adaptive trait that humans and songbirds share. Thus, based on these data, we can infer that the abilities involved in EIP might have set the ground for the emergence of language in humans.

### EIP in Anurans

The adaptive and functional value of EIP emerges quite clearly also considering research on a variety of anurans’ species, which are notably phylogenetically very distant to the *Homo* line. As in humans, and generally, similarly to mammals, the source of vocal sounds in anurans is airflow interruption through vibration of the vocal folds in the larynx (Dudley and Rand, 1991; Prestwich, 1994; Fitch and Hauser, 1995). Calls emitted in different contexts, such as sexual advertisement and male-male aggression, show clear spectral, and acoustic differences (Pettitt et al., 2012; Reichert, 2013). Although it has never been tested empirically, it is plausible that these different call features reflect differences in the level of emotional arousal in the signaler.

In most species of anurans investigated so far, males acoustically compete for females under conditions of high background noise produced by conspecifics. As a consequence, males have developed calling strategies for improving their conspicuousness, i.e., the ability to fine-tune the timing of their calls according to the prosodic and spectral characteristics of the acoustic context (Grafe, 1999).

Anurans aggregate in choruses. The ability for simultaneous acoustic signaling in choruses might have evolved as an anti-predator behavior – specifically, to confuse the predators’ auditory localization abilities (Tuttle and Ryan, 1982) and under sexual selection pressures, as females prefer collective calls to individual male calls. In fact, besides being heard as a group, males have to produce a signal that could stand out from the collective sound in order to attract the female. In order to be heard as a “leader,” advertising individual qualities (Fitch and Hauser, 2003), each signaler has to emit a signal faster than his neighbor. This “time pressure” eventually results in a very tight overlap or synchronization of signals between calling individuals. Females in most species of anurans prefer the calls of “leaders,” individuals that emit more prominent calls (Klump and Gerhardt, 1992), flexibly adjusting their onsets accordingly. Evidence suggests that females in the Afrotropical species *Kassina fusca* prefer leading male calls when the degree of call overlap with the other signallers is high (75 and 90%). However, intriguingly, in this species, females prefer follower male calls when the degree of call overlap is low (10 and 25%). Thus, follower males in *K. fusca* actively adjust their overlap timing in accordance to their vocalizing neighbors, in order to attract females (Grafe, 1999). Ryan et al. (1981) found that in the neotropical frog *Physalaemus pustulosus*, singing in a chorus is adaptive as it decreases the risk of being attacked by a predator and at the same time, increases mating opportunities.

Antiphonal calling in anurans has never been reported. In contrast, duets are described in: the Neotropical *Caphiopus bombifrons* and *Pternohyla fodiens* (Bogert, 1960), the common Mexican treefrogs, *Smilisca baudinii* and in the genuses *Eleutherodactylus* and *Phyllobates* (Duellman, 1967). Tobias et al. (1998) reported remarkable duetting behaviors in the South African clawed frog, *Xenopus* laevis. Females in this species have a very short sexual receptivity time window, in which they have to accurately locate a potential sexual mate. This is not an easy task, considering the high population density and the low visibility in their natural habitat. These constraints may have led to fertility advertisement call by females (rapping) when oviposition is imminent. Tobias et al. (1998) found that females swim to an advertising male and produce the rapping call, which stimulates male approach and elicits an answer call. Thus, the two sexes respond to each other’s calls (which partially overlap), a behavior that results in a rapping–answer interaction. Interestingly, Bosch and Márquez (2001) found that in midwife toads, *Alytes obstetricans*, males engage in duets in competitive contexts. This research suggests that, when duetting, males adjust the temporal structure of their calls, increasing calling rate. This variations correlates with the caller’s body size and seems to affect females’ mate choice.

### EIP In Insects

Crucial implications for the understanding of EIP in humans may derive from research on insects. Notably, this animal taxon is phylogenetically quite distant to humans. Therefore, comparative work on EIP in humans and insects is a perfect candidate to highlight selective pressures underlying the ability to process the prosodic modulation of sounds marking emotional expression and interactional coordination.

It is worth remarking that the mechanisms underlying sound production in insects are extremely different than the ones possessed by the animal taxa reviewed so far. In fact, insects produce advertising or aggressive sounds through stridulation, i.e., vibration of a specific sound source generating by rubbing two body structures against each other, for instance, the forewings in crickets and katydids, or the legs across a sclerotized plectrum, in grasshoppers (Prestwich, 1994; Bennet-Clark, 1999; Hartbauer and Römer, 2016). In the *Expression of the emotions in man and animals*, Darwin (1872) observed that although stridulation is generally used to emit a sexual advertisement signal, bees may vary the degree of stridulation to express different emotional intensities. However, to my knowledge, the auditory expression of emotional arousal in insects has received only little empirically investigation to date (Brüggemeier et al., submitted; Rezával et al., 2016). In contrast, much research on this class of animals has addressed the ability for interactional coordination in sound production.

As to the study of inter-individual coordination as an adaptive analogous trait in humans and insects, it is important to refer to a striking phenomenon in the visual domain: fireflies, winged beetles in the family of *Lampyridae*, use their ability for bioluminescence in courtship or mating contexts (Greenfield, 2005; Ravignani et al., 2014a). Several species of this family are able to entrain in highly precise synchronized flashing, probably to create a more prominent signal to potential mates from a remote location (Buck and Buck, 1966).

Similarly to the case of bioluminescent signals in fireflies, several species of insects have the ability to coordinate timing patterns of their acoustic signals. Specifically, male individuals tend to synchronize their signal within choruses. In ratter ants (genus: *Camponotus*), the ability to entrain in synchronized signal production has evolved as an anti-predator behavior (Merker et al., 2009). However, in most species of studied insects, this ability seems to have evolved under sexual selection pressures (Alexander, 1975; Greenfield, 1994a,b; Yoshida and Okanoya, 2005; Ravignani et al., 2014a). Typically, only males generate acoustic signals, and the mute females approach the singing males. To produce a louder signal that has a better chance to be heard by (and attract) females from a greater distance, advertising males of the tropical katydid species *Mecopoda elongata* tend to synchronize the production of acoustic sounds (Hartbauer and Römer, 2016). Synchrony maximizes the peak signal amplitude of group display, an emergent property known as the “beacon effect” (Buck and Buck, 1966).

In the Neotropical katydid *Neoconocephalus spiza*, females display a strong preference for males that produce a signal after a slight lag, or alternatively, to coincide with, but slightly lead, the other males (Greenfield and Roizen, 1993). As for anurans, male insects have to produce prominent signals to stand out from the group and attract a sexual mate. In the *M. elongata*, in order to lead the chorus, thus being heard by the female, each signaler has to emit a signal before another individual, and at a higher amplitude (Hartbauer et al., 2014). Thus, each male’s emission rate becomes increasingly faster, resulting in synchronization of signals. This suggests that time-coordinated (in this case, synchronized) collective signal is an epiphenomenon created by competitive interactions between males within sexual advertisement contexts (Greenfield and Roizen, 1993). Sismondo (1990) has shown that in *M. elongata*, the dynamic of sound production between leaders and followers has oscillator properties, a finding that echoes data from research on turn-taking dynamics in human conversations.

Antiphonal calling in insects has never been reported. Nonetheless, in multiple orders of insects, individuals of opposite sex engage in time-coordinated duets initiated by the male, with the female replying within a time window that is often species-specific (Zimmermann et al., 1989; Bailey, 2003). Males initiating a duet often insert a trigger pulse at the conclusion of their call, and the females might use this as a cue to which they may reply (Bailey and Field, 2000). Bailey (2003) hypothesized that, in duetting species, females evolved the ability to reply to males to counterbalance predation risk and energy consumption linked to the production of complex and long sounds in males. The author suggested that signal prominence decrease as a result of a counter-selection pressure from male costs.

## EIP in Language Acquisition

As detailed in the previous sections, much research reports on the ability for EIP across a diverse range of animal taxa providing data on both homologous and analogous traits involved in EIP, thus on their adaptive and functional value. These data, combined with evidence on the pervasive use of prosodic modulation of the voice in linguistic communications in modern humans, support the hypothesis that the ability to process EIP might have evolved into the human ability to process linguistic prosody. This holds true not only on a phylogenetic scale, but also for human language development, i.e., on an ontogenetic scale.

When talking to infants, parents of different languages and cultures typically use vocal patterns that are distinct from speech directed at adults: this special kind of speech, commonly referred to as infant-directed speech (hereafter IDS), is often characterized by shorter utterances, longer pauses, higher pitch, exaggerated intonational contours (Fernald and Simon, 1984; Fernald et al., 1989) and expanded vowel space (Kuhl, 1997; de Boer, 2005). IDS is a good example for the ontogenetic role of EIP in humans, with striking effects both on children’s acquisition of language and their development of social cognition. Recent research suggests that caregivers across multiple cultures instinctively adjust their speech prosodic features to their infants (Kitamura et al., 2001; Burnham et al., 2002).

As Fernald (1992) observes, by intuitively moving to a pitch range that an infant is more sensitive to (i.e., where the perceived loudness of the signal is increased), mothers compensate for the infants’ auditory limitations. Indeed, it has been shown that infants’ threshold of auditory brainstem responses (ABR) are higher by 3–25 dB than adult ABR thresholds (Sininger et al., 1997). Given that neonates have greater auditory limitations than adults (Schneider et al., 1979), the speech addressed to neonates needs to be more intense in order to be effectively perceived. A sound of 500 Hz has a higher frequency and will be perceived by the human hearing system as louder than a sound at 150 Hz with the same intensity. It follows that speech with a higher frequency will be more salient to the infant. Therefore, frequency changes seem to be particularly salient: infants tested in an operant auditory preference procedure showed a strong listening preference for the frequency contours of IDS, but not for other associated patterns such as amplitude or duration (Fernald and Kuhl, 1987; Cooper et al., 1997).

The prosodic features typical of IDS modulate the infants’ attention and emotional engagement (Fernald and Simon, 1984; Locke, 1995), scaffolding language development. The specific acoustic parameters used in IDS are very effective in communicating prohibition, approval, comfort, and attention bid (Papoušek et al., 1990; Fernald, 1992; Bryant and Barrett, 2007) and also in conveying emotional content such as love, fear, and surprise (Trainor et al., 2000). Therefore, sound modulation typical of IDS elicits attention and emotional responses in the infants, and conveys crucial information about the speaker’s communicative intent (Fernald, 1989). In addition, the exaggerated pitch parameter cross-culturally employed in IDS provides markers that have the following uses: (a) to highlight target words (Grieser and Kuhl, 1988; Fernald and Mazzie, 1991), (b) to convey language-specific phonological information (Burnham et al., 2002; Kuhl, 2004), (c) as cues to word learning (Thiessen et al., 2005; Filippi et al., 2014), or (d) as cues to the syntactic structure of sentences (Sherrod et al., 1977; Fernald and McRoberts, 1996).

Crucially, caregivers combine sounds and modulate the intonation (frequency, tempo, and amplitude) of speech, engaging in time-coordinated vocal interactions with the children. Contingent responsiveness from caregivers, thus interactive coordination, facilitates language learning (Goldstein et al., 2003; Kuhl et al., 2003; Gros-Louis et al., 2006; Goldstein and Schwade, 2008; Rasilo et al., 2013), and improves the child’s accuracy in speech production. Moreover, caregiver-child interactional coordination scaffolds the child’s social development (Todd and Palmer, 1968; Fernald et al., 1989; Goldstein et al., 2003; Goldstein and Schwade, 2008; Brandt et al., 2012), and her/his acquisition of social conventions, such as turn-taking in conversations (Weisberg, 1963; Kuhl, 1997; Jaffe et al., 2001). Keitel et al. (2013) found that 3-year-old children strongly rely on prosodic information to process conversational turn-taking. Thus, prosodic intonation, in combination with lexico-syntactic information is used by adults and infants as cues to anticipate upcoming turn transitions (Lammertink et al., 2015). In summary, a number of studies indicate that IDS promotes the social and emotional development of infants and favors the acquisition of language. Based on these findings, we can conclude that IDS constitutes a relevant biological signal (Fernald, 1992).

Bringing together comparative data on caregiver-infant communication in humans and chimpanzees with paleo anthropological evidence, Falk (2004) suggested that it is very likely that the first forms of IDS in the early hominins evolved as the trend for enlarging brain size, which made parturition increasingly difficult. This caused a selective shift toward females that gave birth to neonates with relatively small and underdeveloped brains who were, consequently, strongly dependent on caretakers for survival. According to this hypothesis, humans started to make use of prosodic modulations in order to engage infants’ attention, and to convey affective messages to them while engaging in other activities. Interestingly, this would explain why humans are the only species where tutors exaggerate the prosodic features of the signal when speaking to immature offspring. Based on this research, I propose that the use of prosody for emotional communication and interactional coordination was critical for the evolutionary emergence of the first vocalizations in humans. EIP can thus be considered a critical biological ability adopted by humans on both a phylogenetic and an ontogenetic scale.

## Cognitive Link Between Linguistic Prosody and Music: Is EIP their Evolutionary Common Ground?

Music is a universal ability performed in all human cultures (Honing et al., 2015; Trehub et al., 2015) and has often been identified as an evolutionary precursor of language (Brown, 2001; Mithen, 2005; Fitch, 2006, 2010, 2012; Patel, 2006). A number of studies hypothesize that the musical abilities attested in different species of animals constitute homologous or analogous traits, which paved the evolution of language in humans (Geissmann, 2000a; Marler, 2000; Fitch, 2005; Berwick et al., 2011). This line of research follows up on Darwin’s hypothesis on the musical origins of language (see section: “Darwin’s Hypothesis: In the Beginning Was the Song”).

The studies on EIP across animal taxa reviewed in the previous sections, taken together, have crucial implications for this line of research on the origins of language: it is plausible that the ability for EIP evolved into the ability to process linguistic prosody (namely prosodic cues to lexical units, syntactic structure, and discourse structure comprehension), and perhaps also into the ability for music itself. If this is true, than shared traits between the human abilities for linguistic prosody and music should be empirically observable. Indeed, multiple studies show a large overlap between these two domains. Koelsch (2012) suggested that music and language can be positioned along a continuum in which the boundary distinguishing one from the other is quite blurry (Jackendoff, 2009; Patel, 2010). Two interesting cases in which the ability to process linguistic prosody overlaps with music are the so-called talking drums and the whistled languages (Meyer, 2004): the talking drums are instruments whose frequencies can be modulated to mimic tone and prosody of human spoken languages. Whistled language speakers use whistles to emulate the tones or vowel formants of their natural language, keeping its prosody contours (Remez et al., 1981), as well as its full lexical and syntactic information (Carreiras et al., 2005; Güntürkün et al., 2015). Intriguingly, although left-hemisphere superiority has been reported for atonal and tonal languages, click consonants, writing, and sign languages (Best and Avery, 1999; Levänen et al., 2001; Marsolek and Deason, 2007; Gu et al., 2013), recent brain studies (Carreiras et al., 2005; Güntürkün et al., 2015) suggest that whistled language comprehension relies on symmetric hemispheric activation. In addition, empirical evidence from brain imaging research indicates that the ability to process prosodic variations in language plays a vital role in the comprehension of both verbal and musical expressions. For instance, amusic subjects show deficits in fine-grained perception of pitch (Peretz and Hyde, 2003), failing to distinguish a question from a statement solely on the basis of changes in pitch direction (Patel et al., 2008; Liu et al., 2010). This observed difficulty in a sample of amusic patients supports the hypothesis that music and prosody share specific neural resources for processing pitch patterns (Ayotte et al., 2002). Further brain imaging studies report a considerable overlap in the brain areas involved in the perception of pitch and rhythm patterns in words and songs (Zatorre et al., 2002; Patel, 2003; Merill et al., 2012), and in sound patterns processing in melodies and linguistic phrases (Brown et al., 2006). Therefore, based on the outcome of this line of research, we can conclude that the abilities underpinning linguistic prosody and music share cognitive and neural resources. However, is it plausible to identify in EIP an evolutionary common ground for both abilities?

To date, the cognitive and evolutionary link between the ability to process prosody as cue to the emotional state of the signaler and the ability to use prosody as guide to word recognition, or to syntactic and discourse structure, remains open to empirical investigation. In contrast much research has examined the cognitive link between the ability to process emotional prosody and music in humans, showing that in both music and language, specific emotions (e.g., happiness, sadness, fear, or anger) are expressed through similar patterns of pitch, tempo, and intensity (Scherer, 1995; Juslin and Laukka, 2003; Fritz et al., 2009; Bowling et al., 2012; Cheng et al., 2012). For instance, in both channels, happiness is expressed by fast speech rate/tempo, medium-high voice intensity/sound level, medium-high frequency energy, high F0/pitch level, much F0/pitch variability, rising F0/pitch contour, fast voice onsets/tone attacks (Juslin and Laukka, 2003). Research on this topic suggests that musical melodies and emotional prosody are two channels that use the same acoustic code for expressing emotional and affective content.

As to the evolutionary link between the ability to use prosodic cues to coordinated interactions in auditory communication and social entrainment in music, studies conducted on humans suggests that a strong motivation to engage in frames of coordinated activities such as social entrainment or synchronization, favor adaptive behaviors, and specifically, the inclination to cooperate (Hagen and Bryant, 2003; Wiltermuth and Heath, 2009; Kirschner and Tomasello, 2010; Koelsch, 2013; Manson et al., 2013; Morley, 2013; Launay et al., 2014; Tarr et al., 2014). Consistent with these findings, Phillips-Silver et al. (2010) suggest that the ability for coordinated rhythmic movement, and thus entrainment, applies to music and dance as well as to other socially coordinated activities. From their perspective, the ability for music and dance might be rooted in a broader ability for social entrainment to rhythmic signals, which spans across communicative domains and animal species.

Social engagement in time-coordinated activities, as interactive communications or music – promotes prosocial behaviors (Cirelli et al., 2014; Ravignani, 2015). These adaptive behaviors might have favored the evolution of language, including the ability to process and exchange prosodically modulated linguistic utterances within coordinated interactions – on a phylogenetic scale (Noble, 1999; Smith, 2010).

Crucially, in line with this hypothesis, recent findings suggest that social coordination favors word learning also in modern human adults (Verga et al., 2015). Within this frame of research, empirical evidence indicates that children with communicative disorders benefit from music therapy for social skills such as initiative, response, vocalization within an interactive frame of communication (Müller and Warwick, 1993; Bunt and Marston-Wyld, 1995; Elefant, 2002; Oldfield et al., 2003). These findings are consistent with comparative work on the brain neuroanatomy in humans and birds suggesting that social motivation and affect played a key role in the emergence of language at both a developmental and a phylogenetic scale (Syal and Finlay, 2011).

Taken together, these studies point to the existence of a biologically rooted link between (i) the ability to use prosody for the expression of emotions, interactional coordination between multiple individuals, and language processing, and (ii) the ability to process music. However, the hypothesis that the ability for EIP played a crucial role in the emergence of a fully-blown linguistic and musical abilities in humans is currently open to empirical investigation (Bryant, 2013).

## Conclusions

Theories on the origins of language often identify the musical aspect of speech as a critical component that might have favored, or perhaps triggered, its emergence (Rousseau, 1781; Darwin, 1871; Jespersen, 1922; Livingstone, 1973; Richman, 1993; Brown, 2000; Merker, 2000). Indeed, evidence of shared cognitive processes in music and human language has led to the hypothesis that these two faculties were intertwined during their evolution (Brown, 2001; Mithen, 2005; Fitch, 2006, 2010, 2012; Patel, 2006). Crucially, multiple studies have identified musical behaviors shown in different species of animals (Geissmann, 2000a; Marler, 2000; Fitch, 2005; Berwick et al., 2011), as precursors for the evolution of language.

However, in this article I proposed to address the focus of research on language evolution and development toward the ability to process prosody for emotional communication and interactional coordination. This ability, which is widespread across animal taxa, might have evolved into the ability to process prosodic modulation of the voice as cue to language processing, and perhaps also into the biological inclination to music. In support of this hypothesis, I reviewed a number of studies reporting adaptive uses of EIP in non-human animals, where it evolved as anti-predator defense, social development, sexual advertisement, territory defense, and group cohesion. Based on these studies, we can infer that EIP provided the same adaptive advantages to early hominins (Pisanski et al., 2016). In addition, I reviewed research pointing to the processes involved in EIP as common evolutionary traits grounding the abilities to process linguistic prosody and music.

In the course of speech evolution, an increased control of pitch contour might have enabled a greater vocal versatility and expressiveness, building on the limited pitch-control used for emotive, social vocalizations already in use amongst higher primates (Morley, 2013).

This hypothesis is consistent with the “prosodic protolanguage” version of Darwin’s musical protolanguage suggested by Fitch (2005). According to this model, the first linguistic utterances produced by humans, similar to birdsong, were internally complex, lacked propositional meaning, but could be learned and culturally transmitted. The prosodic protolanguage hypothesis harmonizes with the “holistic protolanguage” model (Jespersen, 1922; Wray, 1998), according to which early humans modulated the prosodic values of their vocalizations, conveying messages as whole utterances that were strongly dependent on the context of use. By this model, this first stage was then followed by a process of gradual fractionation of these holistic, prosodically modulated units into smaller items. It is plausible that this process paved the emergence of propositions ruled by combinatorial principles that would increase their learnability, thus the possibility of their cultural transmission (Kirby et al., 2008; Verhoef, 2012). The identification of the cognitive mechanisms underlying EIP has implications for our understanding of the processes involved in the production and perception of such songbird-like protolanguage, thus of the evolutionary process that led to language.

The beneficial value of EIP is evident in modern humans, particularly in the case of speech addressed to preverbal infants, where it favors the developmental process of language learning and emotional bonding. The comparative studies reviewed in this paper indicate that the prosodic modulation of sounds within an interactive and emotion-related dynamic is a critical ability that might have favored the evolution of spoken language (aiding emotion processing, group coordination, and social bonding), and continues to play a striking role in the acquisition of language in humans (Syal and Finlay, 2011). Further empirical research is required to analyze how the ability to modulate prosody for emotional communication and interactional coordination favors the production and perception of the constitutive building blocks of language (phonemes and morphemes) and of the syntactic connections between words or phrases. This line of research might be conducted on infants, by investigating the developmental benefits of EIP on language processing.

Comparative studies have addressed the ability to process linguistic prosody, e.g., trochaic vs. iambic stress patterns in non-human animals (Ramus et al., 2000; Toro et al., 2003; Yip, 2006; Naoi et al., 2012; de la Mora et al., 2013; Spierings and ten Cate, 2014; Hoeschele and Fitch, 2016; Toro and Hoeschele, submitted). Moreover, research has examined non-human animals’ ability to perceive or produce phonemes (Bowling and Fitch, 2015; Kriengwatana et al., 2015). Nonetheless, to my knowledge, the effect of EIP on the perception of the building blocks of heterospecific or conspecific communication systems in non-human animals is still open to empirical examination.

The integration of these studies within a research framework focused on the functional valence of prosodic modulation of the voice in animals, i.e., to its emotional, motivational, and socially coordinated dimensions – will favor a deeper understanding of the evolutionary roots of human emotional and linguistic interactions (Anderson and Adolphs, 2014). Additionally, comparative research on non-human animals and pre-verbal infants, combined with new methods to explore emotional and interactive sound modulation in music and language from a neural and behavioral perspective, promise empirical, and theoretical progress. This investigative framework may ultimately result into new empirical questions targeted at a deeper understanding of the inter-individual, multimodal dimension of communication.

## Author Contributions

The author confirms being the sole contributor of this work and approved it for publication.

## Conflict of Interest Statement

The author declares that the research was conducted in the absence of any commercial or financial relationships that could be construed as a potential conflict of interest.
